# Food Security in Ghanaian Urban Cities: A Scoping Review of the Literature

**DOI:** 10.3390/nu13103615

**Published:** 2021-10-15

**Authors:** Robert Akparibo, Richmond Nii Okai Aryeetey, Evans Atiah Asamane, Hibbah Arabah Osei-Kwasi, Elysa Ioannou, Gisele Infield Solar, Vicki Cormie, Kingsley Kwadwo Pereko, Francis Kweku Amagloh, Samantha J. Caton, Joanne E. Cecil

**Affiliations:** 1 School of Health and Related Research, The University of Sheffield, Sheffield S10 2TN, UK; elysa1996@gmail.com (E.I.); s.caton@sheffield.ac.uk (S.J.C.); 2 School of Public Health, University of Ghana, Accra, Ghana; raryeetey@ug.edu.gh; 3 Institute of Applied Health, University of Birmingham, Birmingham B15 2TT, UK; E.A.Asamane@bham.ac.uk; 4 Geography Department, The University of Sheffield, Sheffield S10 2TN, UK; h.osei-kwasi@sheffield.ac.uk; 5 Sports and Physical Activity Research Centre, Sheffield Hallam University, Sheffield S1 1WB, UK; 6 School of Medicine, The University of Sheffield, Sheffield S10 2TN, UK; ginfieldsolar1@sheffield.ac.uk; 7 University Library, University of St Andrews, St Andrews KY16 9AJ, UK; vhc1@st-andrews.ac.uk; 8 School of Medicine, University Cape Coast, Cape Coast, Ghana; k.pereko@uccsms.edu.gh; 9 Food Science and Technology Department, University for Development Studies, Tamale, Ghana; fkamagloh@uds.edu.gh; 10 School of Medicine, University of St Andrews, St Andrews KY16 9AJ, UK

**Keywords:** food quality, food access, food utilization, food security, urban Ghana, scoping review, urbanization, urban and peri-urban agriculture

## Abstract

Urbanisation in Ghana could be negatively impacting the state of food security, especially in economically vulnerable groups. Food supply, safety, and quality are all aspects of food security which could be impacted. We conducted a scoping literature review to understand the nature and magnitude of evidence available on the urban food security situation in Ghana. A literature search was conducted in Medline, CINAHL, Embase, Global Health, Scopus, Web of Science, Africa Wide Information and Google Scholar to identify relevant peer-reviewed and grey literature. 45 studies, mainly cross-sectional surveys/food samples analysis, met the inclusion criteria. The majority of studies were concentrated in the Greater Accra Region (*n* = 24). Most studies focused on food safety and quality (*n* = 31). Studies on supply and stability were, however, scarce. Qualitative research methods were uncommon in the included studies. The existing literature on food security are concentrated in two regions: The Greater Accra and Ashanti regions. Future studies exploring food security in urban Ghana should focus on exploring the lived experiences and perceptions of food insecurity and food stability by urban-dwellers using qualitative methods. The evidence suggesting that the safety/quality of foods sold in Ghanaian markets is poor should be a concern to consumers and policy makers.

## 1. Introduction

Currently, more than half of the Ghanaian population lives in urban areas [1]. Living in urban areas increases access to income-generating opportunities as well as infrastructure and services that improve quality of living. Such services may include potable water, electricity, health care, schooling, information communication technologies, and paved roads, among others [2]. However, rapid and unplanned urbanization can adversely affect human health and wellbeing. Without appropriate planning and interventions, urban areas can quickly become slums where a combination of poverty, inadequate and unsafe housing, and limited opportunities to access basic services can affect residents ability to access basic necessities of life, including availability, and access to quality food and water [1]. Ghana is increasingly becoming urbanized, as more people migrate to city settings [3]. Therefore, interventions are required to prevent these adverse responses from happening in urban areas in Ghana.

Whenever there is unreliable physical and economic access to sufficient, safe, and nutritious food for all people, a situation of food insecurity, if not existing already, is imminent [4]. The effects of food insecurity are felt across all age groups, although young children, and women of reproductive age experience the most challenging effects of food insecurity [4,5]. Food insecurity is relatively higher in rural settings, compared to urban areas in Ghana [5]. However, it is becoming increasingly realized that urban dwellers, especially the most economically vulnerable population groups (including those living in slums), have specific challenges to food access, quality, and safety, in ways that increase their vulnerability to becoming food insecure and, ultimately experiencing malnutrition [6]. Therefore, focusing food security research efforts on vulnerable urban populations is necessary.

Key issues of interest regarding urban food security are adequacy and sustainability of food supply, food price and affordability, food safety and quality, vulnerabilities, and the capacity to mitigate them. Food supply in urban areas is dependent on interactions across several complex structural and behavioural determinants [1]. As more people move into cities, the farming workforce reduces, and there is loss of arable land located in or near urban areas. This land, instead, is rapidly converted for residential and industrial purposes. This situation can contribute to increased cost of food, due to reduced food supply capacity [7]. High population density and increased demand further exerts pressure on food supply systems, leading to increased food prices. Food prices can also be affected by poor quality infrastructure such as roads linking rural growing communities to urban areas, and cost of transportation to markets. Food prices and supply are, therefore, key issues for urban food security [7].

Low-income families are particularly vulnerable to shocks and situations that influence their ability to generate or save income. Increased food costs due to urbanisation, and also for the reasons described above, are therefore more likely to reduce food security in low-income families. There is also a growing concern about food losses and waste [8,9], which has remained a global challenge [10]; more recently the United Nations has estimated that each year, about 14 percent of the world’s food is lost even before reaching the market [11]. This further exacerbates the problem of food insecurity [10,12,13], especially in the developing world [14]

To be able to cope with food insecurity, urban-dwelling households may adopt less desirable, unhealthy, and unsustainable coping mechanisms such as purchasing food of lower quality or skipping meals [15]. In these coping mechanisms, quality is often traded for quantity, compromising dietary adequacy, food safety and health. Low food quality may also expose consumers in urban settings to food safety risks. Given this, it is imperative to design evidence-informed interventions to address food insecurity to improve dietary quality and safety among urban dwellers; ultimately, this will improve health and wellbeing and prevent potential illness in urban settings.

Lifestyles in urban areas are also associated with increased demand for convenience foods [16]. High-paced work routines that often characterise urban living, increase the likelihood of relying on prepared, processed, pre-packaged, and/or ready-to-eat foods [17]. While these foods are convenient, they are often energy-dense and low in nutrients. Continued exposure to such foods increases risk of diet-related non-communicable diseases (NCDs) [17]. Findings from two case studies in Accra, Ghana and Nairobi, Kenya have demonstrated that urban dwellers prefer diverse diets [17]. However, this preference for diverse diets is limited by the high cost of nutrient-rich foods, pervasive availability and exposure to marketing of processed foods, and easy access to inexpensive unhealthy foods which are often vendored in unhealthy environments [18]. The current Coronavirus (COVID-19) pandemic and the local and global response to address it have magnified pre-existing food insecurity burdens relating to food availability and access in vulnerable communities within urban settings [19].

Several studies in Ghana have highlighted the need for multi-sector interventions to address the complex interactions between food systems, and how they affect urban-dwellers’ nutrition (e.g., [18,20,21,22]). However, currently, there is limited evidence on the scale of the food insecurity situation in urban Ghana, especially evidence around food supply, food access, safety/quality of food, and the utilization of foods among urban dwelling Ghanaians. A better understanding of the available evidence is needed to guide future research, and inform the development of context-specific interventions, in order to improve the food security situation in urban Ghana [23]. For this reason, this scoping review was conducted with the aim to identify and describe the literature on urban households’ food security in Ghana, focusing on food supply, access, utilization, safety and quality.

## 2. Materials and Methods

### 2.1. Design and Setting

A scoping review of the literature on the urban food security research situation in Ghana was conducted. Daudt et al. [24] describe a scoping review as a ‘type of research synthesis that aims to map the literature on a particular topic or research area, and provide an opportunity to identify key concepts; gaps in the research; and types and sources of evidence to inform practice, policymaking, and research’. This type of review is different from a systematic review, which aims to ‘provide answers to questions from a relatively narrow range of quality assessed studies’ [25]. This scoping review of urban food security in Ghanaian cities is intended as a basis for developing interventions that will aim to address food systems in urban Ghana and subsequently for other urban settings in Sub-Saharan Africa. To the best of our knowledge no scoping review has yet examined the literature on urban households’ food security in urban Ghanaian cities, focusing on food supply, access, utilization, safety and quality.

### 2.2. Framework Guiding this Review

The framework defined by Arksey and O’Malley [25], updated by Colquhoun et al. [26], and further detailed in the Joanna Briggs Institute Reviewers Manual: Scoping Reviews [27] was followed in conducting this scoping review. The framework outlines five key steps to conducting a scoping review: (i) identifying the review question, (ii) identifying relevant studies, (iii) study selection, iv) charting the data, and v) collating, summarising and reporting results. Detailed description of how we applied the framework is presented as follows:

Stage 1: Identifying the review question

In this review, we sought to understand the extent of research conducted on urban food security on Ghana in order to determine the value of undertaking a full systematic review [25]. Thus, our review addressed the key questions “what studies currently exist in relation to urban food security in Ghana?”

Stage 2: Identifying relevant studies

We conducted searches in seven databases MEDLINE, EMBASE, CINAHL, SCOPUS, Web of Science Core Collection, African-wide information, and Global Health, from June to July 2020, with an update search completed in July 2021. Google Scholar and websites of the Ministries of Health, and Food and Agriculture were searched for grey literature. Supplementary Materials Table S1 carried out included checking the reference list of included studies (backward chasing), and citation follow-up (forward chasing). These additional search efforts helped identify additional potentially eligible, published studies which were not captured earlier by the academic databases or grey literature sources. Authors in this field were contacted if full texts papers were not available, as well as to ask them for ongoing studies or any rejected manuscripts relevant to the review. Search terms were selected and developed in collaboration with our information specialist (VC) and used for each data base.

Stage 3: Study selection

All citations were imported into EndNote reference manager and screened for relevance. Two levels of relevant screening that best meet the review inclusion criteria were conducted after removal of duplicates citations. Guided by an inclusion and exclusion criteria (Box 1), three reviewers independently screened all titles and abstracts of the search outputs (HOK, EAA and EI). Discrepancies were resolved by a third reviewer and topic expert (RAO). Full texts of potentially qualified abstracts were downloaded and read by all reviewers independently based on the a priori inclusion criteria (Box 1), to select the papers that best addressed the review questions. To eliminate selection bias, a meeting was held to discuss the full texts which were listed as ‘not relevant’ by reviewers, where consensus was agreed.

Box 1 **Inclusion and exclusion criteria.**

Inclusion criteria

We included studies/reports in the review if they;

- Presents findings measuring the level of food security experience with respect to:
  ○ food supply: includes information on food quantity produced, available within community from multiple vendors in urban settings
  ○ Food access: includes information on ability to obtain food without hindrance in urban settings, e.g., price of food, household ability to afford food.
  ○ Food quality/safety: includes information on quality of food with respect to contamination from heavy metals, microorganisms, plastic leaching, polycyclic aromatic hydrocarbons, visual contamination, unhygienic environment, including perceived poor hygiene.
  ○ Food utilization: includes information on food consumption, food allocation at household level but all in the urban setting
  ○ Food stability: includes information on food availability and access over longer duration, including effect of seasonality of food availability and access
  ○ Perceived food security: includes information on scales for assessing food insecurity, including household food security access scale (HFIAS), food insecurity experience scale (FIES), other measures of perceived and experiences food insecurity as well as coping mechanisms
- Conducted in urban or peri-urban Ghana
- Published in English language
- Are qualitative studies, quantitative studies, mixed method and academic/technical reports
- Available online in full texts version
- Human studies

Studies that did not meet the above criteria were excluded.

Stage 4: Charting the data

Key data that were extracted included study reference details (country and year of study); study setting (urban); study target population or food samples studied (including sample size); study design; aspect of food security studied, outcomes that were measured, and key findings on food security. We coded the data in Microsoft Excel and thereafter, conducted a narrative synthesis. We then mapped the evidence against the food security aspects of interest in each included study. The findings are described and presented according to the key food security themes examined. This review does not address the methodological quality of included studies since that was not an aim of this study [26].

Stage 5: Collating, summarising, and reporting results

Here, we have provided a summary description of the study selection process, a description of the study characteristics, including the geographical distribution of the included studies, the study designs the studies used, the population and/or food sample studied, and the sample size.

## 3. Results of Study Selection

**Figure 1:**
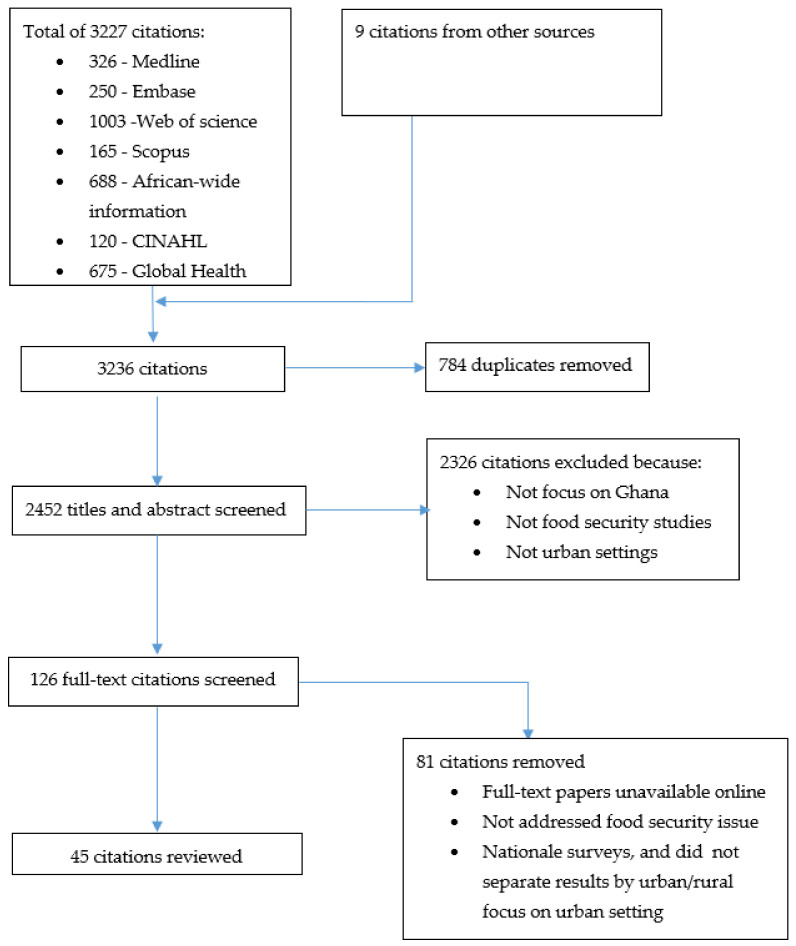
PRISMA flow chart describing the study selection process.

Overall, a total of 3236 citations were retrieved, following the searches that were conducted from all sources. Altogether, 784 duplicates citations were excluded, leaving a total of 2452 papers to be screened. After titles and abstract level screening, 2326 citations were removed. The potentially eligible full texts (*n* = 126) were downloaded and read in full to ascertain their relevance. After this level of screening, 81 citations were excluded, leaving a final set of 45 citations to be included in the review. A summary of the screening process is illustrated in the PRISMA flow chart below (Figure 1).

## 4. Characteristics of the Included Studies

Table 1 summarises the characteristics of the included studies, and the aspect of food security that the studies have focused on. Overall, 45 (all peer-reviewed) studies were included in the review. The year of publication of the documents ranged from 2000 to 2021. The studies were all conducted in urban or peri-urban settings in Ghana. The distribution of the included studies by the geographical regions of Ghana are as follows; twenty-four studies in Greater Accra [20,28,29,30,31,32,33,34,35,36,37,38,39,40,41,42,43,44,45,46,47,48,49,50], eight studies in Ashanti Region [51,52,53,54,55,56,57,58], three studies in the Northern region [22,59,60], and two studies each in the Central region [61,62], Western region [21,63], Eastern [64,65]. Volta [66] and Brong Ahafo [67] regions contributed one study each, while two studies were conducted in two or more regions [49,68]. There were no included studies from the Upper East and Upper West regions. The categories of participants or data units reported in the studies were farmers, street food vendors, restaurant, hotel operators, consumers at household level and on the streets, and foods and vegetables samples (see Table 1). The sample sizes stated in the included studies ranged from 21 (minimum) [20] to 7000 (maximum) [44] human participants, and 6 (minimum) [56] to 3486 (maximum) [36] food samples. Three studies [32,43,62] did not report sample size, and in terms of study designs, all 45 studies were cross-sectional surveys of consumers and or households, and food samples purchased and analysed in the laboratory. 

## 5. Narrative Report of Evidence

### 5.1. Domains of Food Security Studied

Five domains of food security that have been studied and reported in the literature included food access, food supply, food safety and quality, food utilisation, and perceived food security. Of the 45 included studies, food safety and quality were frequently studied (*n* = 31 studies), followed by food access (*n* = 8 studies), and food ulitisation (*n* = 7 studies). Fewer studies on food supply and perceived food insecurity were identified (Table 1). No studies on food stability were identified. The following sections presents the key results according to the aspects of food security area studied, as well as a summary of the evidence which could potentially be useful to Ghanaian policy makers.

### 5.2. Food Access

Food access information was reported in eight studies [21,31,32,50,51,57,61,66]. Two of these studies were conducted in the Ashanti region [51,57], one each in the Western region [21], Central Region [61], Volta region [66], and three in the Greater Accra region [31,32,50]. These studies largely examined the factors that consumers consider as crucial in making decisions to access food from their neighborhood. In the Volta and the Greater Accra regions, cleanliness of the local surroundings, where food is sold, and the hygienic nature of the food handler, plays a key role in consumer choice of food access [50,66]. For instance, one study conducted in the Volta region in Ho [66] examined diners’ decision-making to eat at traditional catering establishments. In this study, factors such as cleanliness of the place, sanitation and hygiene, cleanliness of staff, quality of food, and service staff behaviour were identified as influencers of consumers decisions to access and patronise cooked food sold in their neighbourhoods [66]. Additionally, in the Greater Accra, Central and Western regions, secure food was explained in terms of convenience in accessing food, the availability of food in the local area, and the perceived quality or healthiness of the foods that are available and accessible [21,50,61]. The quantity and cost of the foods were the least worried factors consumers considered, with respect to food access. For examples, in the Central regional capital of Cape Coast, the factors that influenced the respondent’s choice of food and place of eating were the surrounding environments where the food was sold and the cleanliness of the food handlers. Price of the food was less of an issue if the food environment was clean and tidy [61]. On the contrary, two studies from the Ashanti [51] and Western regions [21] revealed that, price of food and vendor’s willingness to offer food on credit influenced consumer food access decision-making.

### 5.3. Food Supply

Food supply to urban areas depends largely on food type and seasonality, and usually arrives from multiple sources. In this review, we described food supply to include information on food quantity produced and is available within community from multiple vendors in urban settings (see inclusion criteria). In our search, we found only two relevant studies that reported on food supply in urbans areas in Ghana [22,50]. One study was conducted in Northern region [22], and the other in the Greater Accra [50]. The study in the Northern region, Karg et al. [22] indicated that most of the food supply in urban areas in Ghana are from small-scale suppliers, usually originating from rural areas. In the Greater Accra region, studies reported that limited food supply was observed in low-income households. For instance, in the study published by Nagai et al. [50], it was revealed that due to high prices of raw foods or their processing cost mothers of low socioeconomic status were unable to acquire/process baby weaning foods, e.g., weanimix—a nutritional meal designed for children who are newly weaned from breast milk—for their newly weaned babies.

### 5.4. Food Datefy and Quality

Food Safety was described, in this study, to include information on quality of food with respect to contamination from heavy metals, microorganisms, plastic leaching, polycyclic aromatic hydrocarbons, visual contamination, unhygienic environment, including perceived poor hygiene. Overall, the review identified 31 studies that focused on food safety and quality issues in urban Ghana. More than half of these studies (*n* = 19) were conducted in the Greater Accra region [16,20,28,32,33,34,35,36,37,38,39,41,42,43,44,45,46,47,48]. The remaining were conducted in the Ashanti region—7 studies [51,52,53,54,55,56,58], Central region—2 studies [61,62], and one study each was conducted in the Northern [59], Western region, [63], and Eastern region [64], and one study conducted in multiple urban Ghanaian cities [68]. In the Greater Accra region, King et al. [28] found that street food vendors did not comply to food safety standards. In this study, 66% of food proprietors surveyed did not obtained meat supply from approved sources. In the majority of studies, food samples including, vegetables and ready-to-eat fruits salads, meat and fish, analysed in the laboratory, were reportedly contaminated with micro-organisms (bacterial, parasites) or heavy metals, above the acceptable levels for consumption [28,33,34,35,36,37,38,42,43,44,45,46,47,48,51,52,53,54,55,56,59,62,63,64]. Kortei et al. [63] analysed ready-to-eat salad in the Western region to determine their quality/safety for consumption, and reported that the mean coliforms and E. coli contamination levels in the salad were 6.35 ± 0.09, and 5.1 ± 0.1 log cfu/g, which were above the acceptable safety level. In the Ashanti region, Akoto et al. [53] revealed that the risk index for combined pesticides due to contamination of all vegetables they analysed was above the acceptable safety standard level. Similar observations about food safety issues in raw foods were made in studies that analysed food and/or vegetable samples sold in various markets in the Greater Accra region [33,34,36,37,38], Eastern region and Western regions [63,64], and also dairy products in the Northern region [59].

### 5.5. Food Utilisation

Food utilisation was reported in seven studies [21,29,31,32,60,67,69]; three in the Greater Accra Region [29,31,32], one each in Northern [60], Western [21] and Brong Ahafo [67] regions. Authors of one these seven studies [69] did not indicate in which specific Ghana region the study was conducted. One of these studies (unspecific region) reported that skipping meals was common among the older age groups (50–64 years) compared to young adults or children [69]. In this study, 36% of the elderly were reportedly skipping meals because of lack of food in their households. In the study conducted in the Western region, the authors reported that energy-dense street foods were more frequently purchased and consumed by residents [21]. In the Sackey et al. [29] study in the Greater Accra region assessing food security and dietary diversity issues, fish compared to meat consumption was frequently observed. After following the study participants over time, the authors reported that the pattern of fish and meat consumption did not change. More people were consuming fish compared with meat consistently [29]. In another study in the Greater Accra region that examined urban household characteristics and dietary diversity, Dake et al. [32] reported that the low socio-economic class group were consumed less fruits and vegetables compared to their higher economic class counterparts. Similarly, Northern region, Saaka and colleagues [60] reported that women of low household wealth index were found to be 48% less likely to meet the minimum dietary diversity for women (MDD-W).

### 5.6. Perceived Food Insecurity

Perceived food insecurity was scarcely studied, with only five studies reporting on this domain of food security [29,30,49,65,69]. Of these studies, two studies were conducted in the Greater Accra region [29,30], and one from the Eastern region [65]. Two studies [49,69] were conducted in multiple urban cities. The study by Bannor et al. [49] was conducted in four different contrasting cities: The Greater Accra, Bono, Ahafo and Bono East Regions, but the remaining study [69] did not specify the particular urban cities or regions data were collected from. These five studies assessed perceived food insecurity at the household level, using different food security questionnaires/scales, including the Household Food Insecurity Access Scale (HFIAS) [29,30,49,65], the Food Insecurity Experience Scale (FIES) [49], and the Household Food Insecurity Access Prevalence (HFIAP) [30,49]. In the study conducted in the Eastern region, Pobee et al. [65] reported, perceived food insecurity was reported among 23% of households surveyed, with 28% of women aged 18–35 years in these households reportedly suffering from multiple micronutrient deficiencies. In the non-specified urban setting study, involving a sample of 1200 individuals aged ≥50 years [69], the prevalence of perceived food insecurity ranged from moderate to severe. In this study, food insecurity indicators were hunger, skipped meals or late intake of first daily meal. The results from this study show that 36% of urban households in Ghana suffer from hunger, and 29% and 5% skipped meals, and had late intake of daily meals respectively. In contrast, the study reported by Bannor et al. [49], conducted in Greater Accra region, Bono, Ahafo and Bono East regions, that compared perceived food insecurity between Urban Ghana and India, concluded that food insecurity in Ghana appear to be mild with an average food insecurity score of 4.05 for each household. This study surveyed 400 urban households from the four regions, using the HFIAS scale to assess perceived food security. In Accra, food insecurity, reported in Tuholke et al. [30] was prevalent among 70% of households. Only one household reported sourcing food from modern supermarkets and fewer than 3% produce food for consumption through gardening, farming, or fishing.

## 6. Discussion

This scoping review has identified studies that addressed different dimensions of urban household food security in Ghana. We identified 45 studies that explored five food security domains: food access, food supply, food safety and quality, food utilisation and perceived food insecurity. The majority of the studies were conducted in the Greater Accra and Ashanti Regions. This finding is not surprising as most health and nutrition research carried out in the last three decades in Ghana have been concentrated in the major Ghanaian cities, especially Accra (in the Greater Accra Region) and Kumasi (in the Ashanti Region). Two possible explanations could be given for this: First the two regions host the most prominent academic and research institutions in Ghana (the University of Ghana and the Noguchi Memorial Centre for Medical Research, based in Accra, and the Kwame Nkrumah University of Science and Technology in Kumasi and the Kumasi Centre for Research in tropical medicine (KCCRTM), in Kumasi). The existence of these institutions, partly explain implementation of the majority of the studies in these two regions. Secondly, the two regions are the most urbanized in Ghana. This situation thus serves as an important prioritization criterion for understanding challenges of rural-urban migration and its potential impact on food security [70]. Although the review findings, overall, suggest that there is a need to promote further studies on food security in urban settings in Ghana, future research in the Ghanaian context should focus on other regions where food security research is limited. This approach will help deepen our understanding of the urban food security situation in more urban cities in Ghana, and not just a few bigger cities.

Notably, three out of the five food security domains (food safety/quality, food access, and food utilization) have been studied the most, especially food safety/quality (*n* = 31 studies). We did not find any study focusing on food stability; only few studies have examined the other food security domains (See Table 1 for results). There is, thus, a clear need to prioritize these unaddressed domains in future studies. The limited number of studies in these other domains may be due to the focus of our review on the urban environment. It is not unusual that food safety and quality was a major focus of the extant food security literature. However, there were several adverse findings regarding poor food safety, especially those sold in Ghanaian markets, despite existing policies and standards for addressing these food safety issues, in urban areas [70]. For the majority of studies that examined food safety and quality in urban Ghana, the findings were similar across studies and suggest that most foods, including raw vegetables, meat and fish sold in Ghanaian markets have characteristics that make them unsafe for human consumption [28,33,34,35,36,37,38,42,43,44,45,46,47,48,51,52,53,54,55,56,59,62,63,64]. These food safety issues include microbial, chemical, and heavy metal contamination. These findings suggest that the challenge is systemic. The evidence also points to non-compliance with existing standards. These findings suggest the need for urgent enforcement of the existing regulations and standards [71,72]. In terms of the nature of the literature reviewed, the review found that food security issues in Ghana are rarely investigated qualitatively.

All the 45 documents included were quantitative studies, conducted using cross-sectional designs. The lack of qualitative studies addressing food security issues in urban settings in Ghana suggest a need for attention to this aspect of research. Qualitative studies are crucially helpful for us to understand the views and perceptions held by urban residents on food security issues in their household, and the lack of these types of studies call for further studies that adopt a qualitative approach to assessing food security. The numerous quantitative studies identified by this review addressing food security issues in urban Ghana suggest that a systematic review, possibly with meta-analysis, could be conducted, and including other African countries, to critically examine the evidence in detail, and to assess the strength and quality of the evidence. Based on the data extracted for this scoping review, the methodological quality of the included studies is likely to be poor resulting in weak evidence, given that some studies were unclear on providing key information on the study including the study location [49], sampling and detailed procedures. [32,37,43]. There was also inadequate information on recruitment and the specific population studied (e.g., [31,57,58]).

### Strengths and Limitations

A key strength of this scoping review is the use of a robust methodological approach, the Arksy framework [25], to guide the review to understand what has been studied with regards to food security in urban Ghana. The framework allows reviewers to systematically search for relevant literature using a search strategy, select studies for inclusion using an inclusion and exclusion criteria, as well as organise and report the findings. At each stage of the study selection and data extraction process, two reviewers independently performed the tasks. This helped eliminate literature selection bias, and ensure rigor. Further, an information specialist (VC) performed the literature search in all academic and grey literature sources using a robust and comprehensive search strategy. The limitations of the review were that no evaluation of the included studies methodological quality was conducted, and restricting the setting to only urban and peri-urban contexts. Thus, the conclusion (below) drawn from the review are based on the available studies and not their intrinsic quality or strength of the evidence.

## 7. Conclusions

This scoping review has presented a rapid overview of the existing research published on urban household food security situation in Ghana, by reporting data from 45 academic literature/studies that reported on the issue explored. This review has identified that, in urban settings in Ghana, food safety and quality as a key component of food security has been widely studied, and the evidence reported suggesting that the safety/quality of foods sold in Ghanaian markets is poor, and below the Ghanaian and international food safety acceptable standards. These findings are useful in not only informing our understanding of what has been studied in Ghana, in terms of urban food security and where research gaps are, the findings will aid policy formulation, interventions and further research decision making about food security in Ghanaian urban cities. Literature is limited on other key aspects of food security such as food access, supply, and utilization and perceive food insecurity. The existing literature on food security is concentrated in two region—the Greater Accra region and Ashanti regions—and largely quantitative in nature. Future studies exploring food security issues should focus on the other regions of the countries where food insecurity remains a challenge. Qualitative studies are also needed to better understand the important contextual issues affecting food security in urban Ghana. Additionally, future research could explore how Ghanaians living in urbans settings perceive their food security situation, and food stability issues.

## Figures and Tables

**Table 1:** Characteristics of 45 included studies and key results.

Study	Region	Design	Population/Sample	Methods (Data Collection)	Key Findings	Evidence Mapped with the Food Security Domains
King et al., 2000) [28]	Greater Accra	Cross sectional Survey	Chop bar operators (*n* = 160)	Observation and interviews	Street food vendors not compliant to safety standards. About 66% of proprietors surveyed did not obtain their meat supply from an approved source: suggestive of food safety issue	Food safety and quality
Kroll et al., 2019) [57]	Ashanti	Cross sectional Survey	Urban households (*n* = 309)	Interviews using food frequency questionnaires	Widespread prevalence of low-protection diets, with a fairly even distribution of low risk and high risk diets.	Food access
Abass et al. 2016) [51]	Ashanti	Cross sectional Survey	Vegetable farms (*n* = 18)	Analysis of randomly selected vegetable samples, followed by interviews with consumers	Vegetable cultivated highly contaminated with feacal microorganisms (fecal coliforms, E coli). Level of fecal coliforms is function of distance to a river. Influencers of accessing food from the street were cost saving, convenience and eating on credit.	Food access, safety and quality
Hiamey, et al., 2013) [21]	Western	Cross sectional Survey	Random consumers (*n* = 220)	Interviews using stanardard structured questionnaires	Street food consumed frequently; particularly carbohydrate-rich foods. Affordability, convenience and access by credit were important drivers	Food access and utilisation
Karg et al., 2016) [22]	Northern	Cross sectional Survey	Food flow records (*n* = 14,000)	Traffic surveys of food flows on access roads	Food supply to urban areas depends highly on food type and seasonality and arrive from multiple sources. Small-scale suppliers bring in most urban food.	Food supply
Mensah, et al. 2017) [66]	Volta	Cross sectional Survey	Hotel Caterers (*n* = 199)	Interviews using self-administered questionnaires.	Influencers of food access are cleanliness of the place, sanitation and hygiene, cleanliness of staff, quality of food, service staff behaviour. Quantity and price were the least important factors.	Food access
Pobee et al., 2020) [65]	Eastern	Cross sectional Survey	Household (women, *n* = 95)	Interviews using the 18-item Household Food Insecurity Access Scale (HFIAS)	Food insecurity was reported prevalent among 23% of the households. Compared to married women, more unmarried women were food insecure	Perceived food in security
Sackey et al., 2018) [29]	G. Accra	Cross sectional Survey	Households (Men/women, *n* = 152)	Food insecurity was measured using the validated Household Food Insecurity Access Scale (HFIAS)	Consumption patterns of individual food groups did not change over time except within the meat and fish groups. Meat consumption decreased from 30% (baseline) to 23% (3-months) and increased to 43% (6-month visit). Fish consumption increased from 83% (baseline) to 92% (3-months) and decreased to 81% (6-months).	Food utilisation and perceieved food insecurity
Tuholske et al., 2020) [30]	G. Accra	Cross sectional Survey	Household consumers (Men/women, *n* = 668)	Household Food Insecurity was measured using the HFIAS, alongside Household Food Insecurity Access Prevalence (HFIAP) and the Food Consumption Score.	Food insecurity was prevalent among 70% of households. Only one household reported sourcing food from modern supermarkets and fewer than 3% produce food for consumption through gardening, farming, or fishing.	Perceieved food insecurity
Gyasi, et al. 2020) [69]	N.S.	Cross sectional Survey	Household consumers (focusing on adults aged >50 yrs) (*n* = 1219)	Scondary data drawn from the AgeHeaPsyWel-HeaSeeB Study dataset	Skipping breakfast reported as severe food insecurity issue among people 50–64 years. 36% of participants reported being hungry because there was not food at home.	Food utilisation and perceieved food insecurity
Nagai et al., 2009) [50]	G. Accra	Cross sectional Survey	Companies, Retailers, Millers consumers, (*n* = 73)	Interviews using structured Questionnaires	Reason for the lack of availability of weanimix to mothers from lower-income families include low awareness of the product. Processing and retail price also margins wean mix availability.	Food access and supply
Codjoe, et al. 2016) [31]	G. Accra	Cross sectional Survey	Urban poor households (men and women) (*n* = 452)	Secondary data drawn from second round of the Regional Institute for Population Studies (RIPS) EDULINK urban poverty and health study	Households consuming seven different food groups within a week, with a mean diversity score of 6.8. Fruits, milk and dietary produce had low dietary diversity due to high prices, lack of nutritional knowledge (taste and preference).	Food access, and utilisation
Dake et al., 2016) [32]	G. Accra	Cross sectional Survey	Urban poor households (females) (N.S)	Data on the local food environment collected and analysed using geographic positioning system (GPS) technology	The local food environment (convenience stores, abundance of out-of-home cooked foods and limited fruits and vegetables options) suggestive of an obesogenic food environment.	Food access and utilisation
Darko, et al. 2016) [55]	Ashanti	Cross sectional Survey	Raw food samples (*n* = 40)	Interviews using semi-structured questionnaire	Foods tested were above the acceptable levels and could be sources of food borne pathogens, attributed to poor food hygiene and inadequate processing.	Food Safety and quality
Kortei et al., 2020) [63]	Western	Cross sectional Survey	Fish samples (*n* = 16)	Analysis of mandomly purchased fish samples	All the examined fish species had toxic mineral concentrations within the E.U quality standard limits except for Mercury (Hg) which exceeded the set limits of WHO.	Food Safety and quality
Kudah, et al. 2018) [64]	Eastern	Cross sectional Survey	Vegetable salad sample (*n* = 360)	Analysis of randomly purchased fresh vegetables samples	58% of the food tested (vegetables, spring onion and tomatoes) were found to be contaminated with at least one type of parasite.	Food Safety and quality
Kunadu et al., 2019) [20]	G. Accra	Cross sectional Survey	Consumers (*n* = 176), farmers (*n* = 21) & diary product (*n* = 140)	Analysis of randomly purchased samples of diary milk and milk products, followed by questionnaires interviews with consumers	Fecal coliforms in dairy products, such as brukina, wagashi, and yogurt exceeded the specified limit of 10 CFU/mL, while the prevalence of E. coli and K. pneumoniae were 70 and 65%, respectively. Generally, respondents perceived indigenous dairy as unsafe.	Food Safety and quality
Abubakari et al., 2015) [52]	Ashanti	Cross sectional Survey	Cooked food sample (*n* = 170)	Analysis of purchased ready prepared fruit-salad samples	Ready to be eaten salad foods was a food concern. Three samples tested showed positive to E. coli O157:H7. Mean logcfu/g of total coliforms and E. coli were found to be 6.35 ± 0.09 and 5.1 ± 0.1.	Food Safety and quality
Adam, et al. 2014) [61]	Central	Cross sectional Survey	University students (*n* = 1106)	Interviews using structured questionnaires	Students’ concern on food temperature as a food safety issue. Clean and hygienic eating tables as a key food safety concern and predictor of choice of eating place.	Food access, Food Safety and quality
Addo et al., 2007) [33]	G. Accra	Cross sectional Survey	Cooked food sample (*n* = 10)	Analysis of randomly purchased cooked food samples	Food samples contaminated with Bacteria. 35% of food tested were positive for other coliform bacteria. Almost all (70 %) of the swabs positive for coliforms were from either a cutting board or a working surface	Food Safety and quality
Adzitey, et al. 2020) [59]	Northern	Cross sectional Survey	Food sample (*n* = 200)	Analysis of randomly purchased food and diary milk and milk products	Milk products and other food samples tested were found to be contaminated with Salmonella enterica.	Food Safety and quality
(Adzitey, et al., 2018) [34]	G. Accra	Cross sectional Survey	Meat sample (*n* = 32)	Analysis of selected beef and other food samples	Heavy metals were found present in varying concentrations in foods sample, but were below the maximum limit, and so less harmful for consumption	Food Safety and quality
Akoto et al., 2015) [53]	Ashanti	Cross sectional Survey	Vegetable sample (*n* = 20)	Fresh vegetable samples were randomly purchased and analysed using standard methods	Varying degrees of contamination found in Vegetables. Overall risk index for combined pesticides due to consumption of all vegetables was >1 which pose as a health risk.	Food Safety and quality
Egbon 2013) [35]	G. Accra	Cross sectional Survey	Food sellers (*n* = 148)	Fresh legumes samples were randomly purchased and analysed using standard methods.	Cowpea was found to be infested with callosobruchus macculatus and sitophilus oryzae.	Food Safety and quality
Feglo 2012) [56]	Ashanti	Cross sectional Survey	Raw food samples (*n* = 6)	Raw foods were selected randomly and analysed using standard methods	High levels of bacterial contamination at varying degrees detected in food types tested (higher levels of contamination than acceptable reference)	Food Safety and quality
Ayroe et al., 2016) [54]	Ashanti	Cross sectional Survey	Consumers (*n* = 200), and meat samples (*n* = 105)	Analysis of meat samples, followed by questionnaire interviews with meat sellers sampled randonly	High proportion of offals sold in Kumasi contained lesions (abscesses, metazoan parasites and granuloma).	Food Safety and quality
Fosu et al., 2017) [36]	G. Accra	Cross sectional Survey	Fruits & vegetables samples (*n* = 3483)	Fresh fruits and vegetable sampled randomly and analysed using standard methods	Pesticides residues were detected fruits and vegetables tested. Samples tested contained levels above the MRL levels.	Food Safety and quality
(Nyarko, et al., 2011) [37]	G. Accra	Cross sectional Survey	Fish sample (smoked salmon) (N.S)	Smoked fish samples randomly selected and analysed using standard methods	Microbial counts for samples collected from the smoking sites were within acceptable limits of Ghana Standard Board (GSB), while those from marketing centres were not.	Food Safety and quality
(Nyarko, et al., 2011) [62]	Central	Cross sectional Survey	Food sample (tiger nuts) (*n* = 24)	Fresh fruits samples were randomly selected and analysed using standard methods	Tiger nuts (non-sterile) sold in markets in cape coast are contaminated with high bacteria loads that are implicated in both food spoilage and food-borne diseases.	Food Safety and quality
(Obeng et al., 2018) [38]	G. Accra	Cross sectional Survey	Vegetable sample (tomatoes) (*n* = 120)	Raw vegetables samples were randomly selected and analysed using standard methods.	Varying levels of antibiotic resistance bacteria found in tomatoes sold at various markets centres in Ghana.	Food Safety and quality
(Omari, 2018) [39]	G. Accra	Cross sectional Survey	Fast foods sellers (*n* = 425)	Interviews using structured questionnaires	Respondents expressed concerns about food hazards and other food safety issues: pesticide residue in vegetables, excessive use of artificial flavour enhancers and colouring substances, bacterial contamination, leaked harmful substances from plastic packages and general unhygienic conditions under which food is prepared and sold.	Food Safety and quality
(Omari, 2017) [40]	G. Accra	Cross sectional Survey	Fast foods consumers (*n* = 425)	Interviews using structured questionnaires	Consumer concerns about safety of fast foods. Consumers’ perception of safety of fast food influenced by components of trust (institutional competence and openness).	Food Safety and quality
(Mahami 2014) [41]	G. Accra	Cross sectional Survey	Probiotic yoghourts samples (*n* = 20)	Milk and milk products were sampled randomly and analysed using standard methods	About a quarter of imported and local yoghurt samples were below the recommended standard of ≥ 106 CFU/ml. PH values of samples were within the recommended standard of ≤4.5.	Food Safety and quality
(Mensah, 2014) [58]	Ashanti	Cross sectional Survey	Consumers (*n* = 200)	Interviews using structured questionnaires	Consumers concerns about safety and quality of leafy vegetables in the retail market due to excessive use of chemicals and contaminated water for vegetables production. Further concerns expressed regarding mishandling of leafy vegetables in the retail market, which was viewed as a health risk.	Food Safety and quality
(Mensah et al., 2002) [42]	G. Accra	Cross sectional Survey	Food samples (*n* = 117)	Food samples were selected randomly and analysed using standard methods	Mesophilic bacteria were detected in 356 foods (69.7%): 28 contained Bacillus cereus (5.5%), 163 contained Staphylococcus aureus (31.9%) and 172 contained Enterobacteriaceae (33.7%). Most of the foods sample (salads, macaroni, fufu, omo tuo and red pepper) were contaminated with Mesophilic bacteria (Bacillus cereus, Staphylococcus aureus and Enterobacteriaceae) above acceptable levels.	Food Safety and quality
(Otoo 2013) [67]	B. Ahafo	Cross sectional Survey	Mothers/caregivers (*n* = 246)	Interviews using food frequency questionnaires	Children consumed from at least four food groups and Orphans had a higher dietary diversity than non-orphans did.	Food utilisation
(Pesewu et al., 2014) [43]	G. Accra	Cross sectional Survey	Vegetable samples (N.S)	Vegetables were sampled randomly and analysed using standard methods	Food sample tested have high bacterial contamination.	Food Safety and quality
(Sinayobye 2011) [44]	G. Accra	Cross sectional Survey	Food mill operators (*n* = 21) and food samples (*n* = 36)		Food samples were found to be contaminated with microorganisms. The contamination load increased with the milling time	Food Safety and quality
(Soriyi, 2008) [45]	G. Accra	Cross sectional Survey	Beef sample (*n* = 128)	Meat samples were selected randomly and analysed using standard methods	Beef samples were contaminated with Aerobic mesophiles (189-23000 cfu/g), Staphylococcus aureus (22–59 cfu/g), Bacillus cereus (17–41 cfu/g), Clostridium perfringens (21–48 cfu/g) and Escherichia coli (31–2200 cfu/g).	Food Safety and quality
(Quansah et al., 2018) [46]	G. Accra	Cross sectional Survey	Vegetable sample (*n* = 50)	Vegetables were sampled randomly and analysed using standard methods	Food sample tested positive for enterococci and fecal coliform.	Food Safety and quality
(Yeboah-Manu et al., 2010) [47]	G. Accra	Cross sectional Survey	Food samples (*n* = 27)	Foods were sampled randomly and analysed using standard methods.	Slightly above half of the food tested had E. coli values within the acceptable limits whiles 40.7% were outside the limit—unsafe for consumption.	Food Safety and quality
(Kortei et al. 2021) [68]	Multiple urban settings	Cross sectional Survey	Food sample (*n* = 80)	Raw foods sampls selected randomly and analysed using standard methods.	61.25 % of the food sample tested positive for AFB1 and ranged from 0.38 ± 0.04–230.21 ± 22.14 μg/kg, of which 41.25 % of the samples were above the Europeam amd Ghana food safety standards limits.	Food Safety and quality
Olu-Taiwo et al. 2021) [48]	G. Accra	Cross sectional Survey	Meat samples (from *n* = 6 open markets)	Beef samples selected randomly and analysed using standard methods.	Bacterial contamination of retailed beef sold in different Accra markets. Beef samples mostly contaminated with Staphylococcus spp. (34%), Klebsiella oxytoca (17%), Enterobacter spp. (15%), and Proteus vulgaris (3%).	Food Safety and quality
(Saaka et al. 2021) [60]	Northern region	Cross sectional Survey	Women (*n* = 423)	24hr dietary recall of dietary intake/food consumption.	Results showed that women of low household wealth index were 48% less likely meet the minimum dietary diversity for women (MDD-W), whiles those from households of poor food insecurity were 88 % less likely to achieve the MDD-W.	Food utilisation
(Bannor et al. 2020) [49]	G. Accra, Bono, Ahafo and Bono East	Cross sectional Survey	Farmers (*n* = 400)	Food security status of urban households was assessed using the HFIA Scale and Food Insecurity Experience Scale (HFIES)	Households food insecure in Ghana were reportely mildly, with an average food security score of 4.05 for each household	Perceived food security

Notes: F = Farmers, W = Women and MP = milk products, G. = Greater, N.S. = Not Stated.

## Data Availability

Data extracted for the review is available upon request.

## Supplementary Materials

The following are available online at https://www.mdpi.com/article/10.3390/nu13103615/s1. Table S1: Search strategy.
