# Supplementary material for: Food Security in Ghanaian Urban Cities: A Scoping Review of the Literature
Source: Nutrients. 2021 Oct 15;13(10):3615. doi: 10.3390/nu13103615 (PMC8540338; doi:10.3390/nu13103615)
Supplement: Supplementary file 1 [file nutrients-13-03615-s001.zip › nutrients-1375970-supplementary.pdf]

SUPLEMENTARY FILES: Appendix 1. Table S1: Search strategy:

Database	Search strategy	Results
Ovid Medline	1. ("food security" or "food shortag*" or "food suppl*" or "food qualit*" or "food insecurit*" or "food sufficienc*" or "food insufficienc*" or "food availabil*" or "food utiliz*" or "food utilis*" or "food stability" or "food safety" or "food access*" or "food consumpt*" or "food consumer*").mp.<br>2. (Ghana or Tamale or Bolgatanga or Wa or Volta or Ho or Brong Ahafo or Sunyani or Techiman or Ashanti or Kumasi or Accra or Sekondi-Takoradi or Takoradi or Cape Coast or Tema or Koforidua).mp.<br>3. 1 and 2<br>4. limit 3 to english language<br>5. exp animals/ not humans.sh.<br>6. 4 not 5	326
Embase	1. ("food security" or "food shortag*" or "food suppl*" or "food qualit*" or "food insecurit*" or "food sufficienc*" or "food insufficienc*" or "food availabil*" or "food utiliz*" or "food utilis*" or "food stability" or "food safety" or "food access*" or "food consumpt*" or "food consumer*").mp.<br>2. (Ghana or Tamale or Bolgatanga or Wa or Volta or Ho or Brong Ahafo or Sunyani or Techiman or Ashanti or Kumasi or Accra or Sekondi-Takoradi or Takoradi or Cape Coast or Tema or Koforidua).mp.<br>3. 1 and 2<br>4. limit 3 to english language<br>5. 3 not ((exp animal/ or nonhuman/) not exp human/)<br>6. limit 5 to medline<br>7. 5 not 6	250
Web of Science Core Collection	TOPIC:<br>(("food security" OR "food shortag*" OR "food suppl*" OR "food qualit*" OR "food insecurit*" OR "food sufficienc*" OR "food insufficienc*" OR "food availabil*" OR "food utiliz*" OR "food utilis*" OR "food stability" OR "food safety" OR "food access*" OR "food consumpt*" OR "food consumer*"))<br>AND<br><br>TOPIC:<br>((Ghana OR Tamale OR Bolgatanga OR Wa OR Volta OR Ho OR Brong Ahafo OR Sunyani OR OR Techiman Ashanti OR Kumasi OR Accra OR Sekondi-Takoradi OR Takoradi OR Cape Coast OR Tema OR Koforidua))<br>Refined by: LANGUAGES: (ENGLISH)	1003
SCOPUS	((TITLE-ABS-KEY ("food security" OR "food shortag*" OR "food suppl*" OR "food qualit*" OR "food insecurit*" OR "food sufficienc*" OR "food insufficienc*" OR "food availabil*" OR "food utiliz*" OR "food utilis*" OR "food stability" OR "food safety" OR "food access*" OR "food consumpt*" OR "food consumer*")) AND (TITLE-ABS-KEY<br>(ghana OR tamale OR bolgatanga OR wa OR volta OR ho OR brong A ND ahafo OR sunyani OR Techiman OR<br>ashanti OR kumasi OR accra OR sekondi-takoradi OR takoradi OR "Cape Coast" OR tema OR koforidua)) AND (LIMIT-TO<br>(LANGUAGE , "English"))	165

Africa-wide Information	(("food security" OR "food shortag*" OR "food suppl*" OR "food qualit*" OR "food insecurit*" OR "food sufficienc*" OR "food insufficienc*" OR "food availabil*" OR "food utiliz*" OR "food utilis*" OR "food stability" OR "food safety" OR "food access*" OR "food consumpt*" OR "food consumer*") AND (Ghana OR Tamale OR Bolgatanga OR Wa OR Volta OR Ho OR Brong Ahafo OR Sunyani OR Techiman OR Ashanti OR Kumasi OR Accra OR Sekondi-Takoradi OR Takoradi OR Cape Coast OR Tema OR Koforidua)) NOT animals<br>Limited to English	688
CINAHL	(("food security" OR "food shortag*" OR "food suppl*" OR "food qualit*" OR "food insecurit*" OR "food sufficienc*" OR "food insufficienc*" OR "food availabil*" OR "food utiliz*" OR "food utilis*" OR "food stability" OR "food safety" OR "food access*" OR "food consumpt*" OR "food consumer*") AND (Ghana OR Tamale OR Bolgatanga OR Wa OR Volta OR Ho OR Brong Ahafo OR Sunyani OR Techiman OR Ashanti OR Kumasi OR Accra OR Sekondi-Takoradi OR Takoradi OR Cape Coast OR Tema OR Koforidua))<br>Limited to English	120
Global Health (Search run on the 30th June)	("food security" OR "food shortag*" OR "food suppl*" OR "food qualit*" OR "food insecurit*" OR "food sufficienc*" OR "food insufficienc*" OR "food availabil*" OR "food utiliz*" OR "food utilis*" OR "food stability" OR "food safety" OR "food access*" OR "food consumpt*" OR "food consumer*") AND (Ghana OR Tamale OR Bolgatanga OR Wa OR Volta OR Ho OR Brong Ahafo OR Sunyani OR Techiman OR Ashanti OR Kumasi OR Accra OR Sekondi-Takoradi OR Takoradi OR Cape Coast OR Tema OR Koforidua) AND NOT (DE "animals" OR DE "aquatic animals" OR DE "Chordata" OR DE "circus animals" OR DE "desert animals" OR DE "detritivores" OR DE "domestic animals" OR DE "female animals" OR DE "feral animals" OR DE "game animals" OR DE "herbivores" OR DE "invertebrates" OR DE "laboratory animals" OR DE "male animals" OR DE "meat animals" OR DE "newborn animals" OR DE "pets" OR DE "pollinators" OR DE "predators" OR DE "prey" OR DE "racing animals" OR DE "sentinel animals" OR DE "skin producing animals" OR DE "soil fauna" OR DE "stray animals" OR DE "transgenic animals" OR DE "wild animals" OR DE "working animals" OR DE "young animals" OR DE "zoo animals")	675
